# Food-Induced Adverse Reactions: A Review of Physiological Food Quality Control, Mucosal Defense Mechanisms, and Gastrointestinal Physiology

**DOI:** 10.3390/toxics13010061

**Published:** 2025-01-16

**Authors:** Dongdong Guo, Chang Liu, Hongkang Zhu, Yuliang Cheng, Xiang Huo, Yahui Guo, He Qian

**Affiliations:** 1 State Key Laboratory of Food Science and Resources, School of Food Science and Technology, Jiangnan University, Wuxi 214122, China; 2 Key Laboratory of Pathogenic Microorganisms for Emerging and Outbreaks of Major Infectious Diseases, Jiangsu Engineering Research Centre for Health Emergency Response, Jiangsu Provincial Center for Disease Control and Prevention, Nanjing 210009, China

**Keywords:** food quality and safety management, food toxicity and allergic reactions, host immune defense mechanisms, gastrointestinal response mechanisms

## Abstract

Although food is essential for the survival of organisms, it can also trigger a variety of adverse reactions, ranging from nutrient intolerances to celiac disease and food allergies. Food not only contains essential nutrients but also includes numerous substances that may have positive or negative effects on the consuming organism. To protect against potentially harmful components, all animals have evolved defense mechanisms, which are similar to antimicrobial defenses but often come at the cost of the organism’s health. When these defensive responses are exaggerated or misdirected, they can lead to adverse food reactions, where the costs outweigh the benefits. Furthermore, due to the persistent toxicity of harmful food components, the failure of defense mechanisms can also result in pathological effects triggered by food. This article review presents a food quality control framework that aims to clarify how these reactions relate to normal physiological processes. Organisms utilize several systems to coexist with symbiotic microbes, regulate them, and concurrently avoid, expel, or neutralize harmful pathogens. Similarly, food quality control systems allow organisms to absorb necessary nutrients while defending against low-quality or harmful components in food. Although many microbes are lethal in the absence of antimicrobial defenses, diseases related to microbiome dysregulation, such as inflammatory bowel disease, have significantly increased. Antitoxin defenses also come with costs and may fail due to insufficiencies, exaggerations, or misdirected actions, ultimately leading to adverse food reactions. With the changes in human diet and lifestyle, the failure of defense mechanisms has contributed to the rising incidence of food intolerances. This review explores the mechanisms of antitoxin defenses and analyzes how their failure can lead to adverse food reactions, emphasizing the importance of a comprehensive understanding of food quality control mechanisms for developing more effective treatments for food-triggered diseases.

## 1. Introduction

Food-related syndromes, such as food intolerances, irritable bowel syndrome (IBS), food allergies, and celiac disease, have become increasingly prevalent in modern society, with rising incidence rates [1,2]. While some progress has been made in uncovering the proximal mechanisms of these diseases, their underlying causes remain unclear. These diseases have complex etiologies influenced by multiple factors, including genetics, environment, and diet. To better understand these syndromes, it is essential to delve deeper into the potential physiological systems involved in food responses.

In this context, the concept of “food quality” becomes particularly significant. It not only determines the nutritional value that food provides to the organism but also influences the adverse reactions that food may trigger. Food quality is determined by the complex interactions among various components, each of which can have a positive, negative, or neutral impact on the consuming organism [3]. From an ecological perspective, food quality control mechanisms can be viewed as an evolutionary strategy by which animals meet their nutritional needs while avoiding harmful components in food.

Within this framework, the concept of defense mechanisms is crucial. These are physiological and behavioral processes that protect animals from harmful food components, such as plant-derived toxins [4]. We refer to these defense mechanisms as “antitoxin” defenses, which are highly similar to antimicrobial defenses and play a critical role in maintaining health [5]. Antitoxin defenses are essential in protecting animals from the toxic substances inevitably present in natural food. However, just as antimicrobial defenses can be imperfect, dysfunctions, overactivity, or misregulation of antitoxin defenses can lead to a range of pathological conditions, including the diseases we are concerned with.

Furthermore, with changes in human diet and lifestyle, the failure of these defense mechanisms has become increasingly common. The widespread consumption of highly processed foods, overuse of antibiotics, and extensive use of additives and preservatives have contributed to this imbalance in defense mechanisms. In this process, the disruption of gut microbiota has also played a significant role. Dysbiosis, or microbial imbalance, is closely linked to the onset and exacerbation of various food-related diseases, such as irritable bowel syndrome, inflammatory bowel diseases (IBD), and food allergies. Moreover, as pollution, chemical exposure, and dietary habits change, the mismatch between the modern food environment and our evolved defense mechanisms becomes ever more apparent [6,7].

In this review, we propose a novel framework for understanding food-related diseases, focusing on the role of food quality control mechanisms. We will explore how these mechanisms fail in modern environments, leading to a range of food-induced diseases. The central idea is that the defense system plays a crucial role in protecting animals from low-quality food components and pathogenic microorganisms, and its dysregulation may lead to disease [8]. We argue that many symptoms triggered by food are, in fact, manifestations of defense mechanism failure. Although these mechanisms are vital in natural environments, they are prone to maladaptation or overactivation in today’s dietary environment, leading to food-related diseases. A key feature of this model is the emphasis on the dynamic relationship between the host, food, and symbiotic microorganisms. This relationship is not passive but is continually shaped by evolutionary pressures and closely tied to the organism’s perception, assessment, and response to food. The balance between nutritional intake and defense mechanisms is highly fragile; any disruption to this balance, whether through unhealthy diets, microbial dysbiosis, or changes in environmental factors, can have profound impacts on health [9,10].

Additionally, the concept of defense failure, whether due to insufficiency, overreaction, or incorrect activation, offers an important perspective for understanding the pathogenesis of food-related diseases. We believe that many modern food intolerances and allergic reactions arise from this dysregulation of defense mechanisms. These defenses are crucial in natural environments, but in the modern dietary context, where processed foods are prevalent, essential nutrients are lacking, and harmful substances are abundant, their failure has become more evident, leading to the occurrence of adverse food reactions [11].

Therefore, the aim of this review is to provide a more comprehensive understanding of food-related diseases through an in-depth exploration of food quality control mechanisms, particularly focusing on how defense mechanisms fail in modern environments, leading to various adverse reactions. By uncovering these mechanisms, we hope to offer new insights and strategies for the treatment and intervention of food-induced diseases, advancing the development of more effective therapeutic approaches.

## 2. Food Components, Quality Control, and Sensory Evaluation Mechanisms

### 2.1. Nutritional Demands and Adaptive Strategies of Animals

To meet their nutritional requirements, animals must select available food from their environment. However, these food items vary significantly in quality, nutritional composition, and potential hazards. Consequently, animals must employ a series of complex strategies during foraging to ensure they acquire sufficient nutrition while effectively avoiding harmful substances. This adaptive behavior and physiological mechanism is collectively referred to as “nutritional strategy”, which evolves through natural selection and aims to optimize the animal’s survival and reproductive success within its ecological niche [12].

Nutritional strategies can be classified based on the diversity of food choices. Specifically, animals are categorized as either “dietary generalists” or “dietary specialists”. Dietary generalists typically consume a wide range of food types, while dietary specialists tend to rely on a limited number of specific foods. For example, humans are extreme dietary generalists, capable of consuming and digesting a diverse array of food items, while species such as ground squirrels, giraffes, and honeyguides are extreme dietary specialists, depending almost exclusively on eucalyptus leaves, plant seeds, or honey. Broad specialists, such as herbivores and carnivores, rely on a variety of plant- or animal-based foods, albeit within a more restricted range [13].

While nutritional strategies are often observable through an animal’s feeding behavior, a comprehensive understanding requires a deeper exploration of the animal’s physiological adaptations. Key physiological traits, including the anatomical structure of the gastrointestinal system, digestive capacity, and metabolic characteristics, determine the efficiency with which animals extract and absorb nutrients from their food. For instance, certain animals possess specialized digestive systems that allow them to effectively process specific food types and maximize nutrient extraction, enabling them to thrive within their particular dietary range [12].

However, nutritional strategies extend beyond the acquisition of nutrients; they also involve mechanisms for dealing with potential toxins in food. Many food components can be toxic, and even trace amounts may have detrimental effects on health. Therefore, an animal’s nutritional strategy must incorporate “antitoxin defense mechanisms”. These defenses help animals identify and avoid harmful food items or detoxify and excrete toxins through metabolic and elimination processes. For example, certain food components may trigger immune responses in the gut or activate detoxification pathways, thereby reducing the harmful impact of toxic substances [14].

Thus, the complete nutritional strategy of an animal is a multifaceted, dynamically adjusted system of adaptation. It encompasses not only how to obtain adequate nutrition and optimize nutrient absorption but also how to cope with potential toxins in food. This strategy enables animals to survive and reproduce effectively in complex ecological environments and maintain physiological balance in the face of fluctuating food resources [15].

### 2.2. Food Nutritional Components and Quality Regulation

The food consumed daily is a complex mixture composed of macronutrients (such as carbohydrates, proteins, vitamins, and lipids), micronutrients (such as vitamins and minerals), and non-nutrient compounds [16,17]. The relative abundance and composition of these food components determine their positive, negative, or neutral effects on the consuming animals, collectively influencing the intrinsic quality of the food. Furthermore, the impact of food may also be modulated by the animal’s physiological state and the composition of its gut microbiota. In general, when micronutrients and macronutrients are ingested in appropriate amounts and ratios, they are beneficial to health, while excessive or insufficient intake can lead to adverse effects. This principle is validated by Bertrand’s Law, with deficiencies or toxic symptoms of vitamins serving as a clear example. The proportions of food components are critical determinants of food quality [17,18].

Non-nutrient compounds, including indigestible plant components, plant-derived bioactive substances, and artificial food additives, also significantly influence food quality. Some plant-derived compounds support gut function and metabolism. For instance, indigestible fibers aid intestinal motility and can be converted by the gut microbiota into short-chain fatty acids (SCFAs), providing energy for colonic epithelial cells and exhibiting anti-inflammatory properties [19,20]. However, many plant secondary metabolites (PSMs) are toxic, and evolved to deter herbivores from feeding. These compounds can interfere with nutrient absorption or disrupt host physiological functions, potentially causing harm or dysfunction in animals. For example, some plant metabolites inhibit digestive enzymes, affecting nutrient absorption, while others may damage the intestinal epithelial barrier. Additionally, non-nutrient components may undergo chemical modifications during the host’s metabolism, altering their beneficial or harmful effects on the animal. It is crucial to note that the value of food components is not fixed but is influenced by the host’s physiological state. The biological state may vary due to developmental stages, aging, changes in energy requirements, genetic differences, chronic diseases, and environmental factors. Consequently, the nutritional needs and vulnerabilities of different hosts can differ. For instance, during pregnancy, hormonal changes increase a woman’s demand for specific calories and nutrients while also making her more susceptible to infections and toxins.

Building upon the above strategies, animals must respond appropriately to the foods they encounter to optimize nutrient intake. For instance, to obtain sufficient amino acids, animals prioritize selecting protein-rich foods, secrete proteases to digest proteins, and absorb the resulting amino acids. When food contains toxins, animals must activate detoxification and other defense mechanisms to mitigate the harmful effects of these toxins. The ability of animals to make such food-specific responses stems from their complex food perception and evaluation systems, which guide food selection and response strategies. We refer to the collective operation of these mechanisms as “food quality control”, highlighting the critical role of detecting and defending against toxic food components. Examples of harmful effects of PSM on animals are shown in Table 1 [21].

In summary, food quality is the result of the interplay of multiple factors, including the balance of macronutrients and micronutrients, the influence of non-nutrient compounds, and the regulation of the host’s physiological state. Animals’ food selection strategies and response mechanisms aim to ensure adequate nutrition while minimizing the impact of toxins. These adaptive mechanisms form a dynamic and intricate food quality control system, which not only ensures the fulfillment of physiological needs but also provides defense mechanisms against potentially harmful components, thereby optimizing animal survival and health [17].

### 2.3. Food Component Perception and Sensory Evaluation System

To assess the quality of food, animals rely on a variety of sensory mechanisms to detect key components [29]. This process is similar to the immune system’s detection of pathogens through pattern recognition receptors. For example, glucose is detected via the TAS1R2/TAS1R3 receptors, while glutamate is recognized through the TAS1R1/TAS1R3 and mGluR1/mGluR4 receptors [30]. Certain toxic substances can be sensed via bitter taste receptors (TAS2Rs) [31]. However, the sensory system of animals can only directly detect a small fraction of the thousands of food components, many of which include potentially toxic substances. Without appropriate defense mechanisms, animals could ingest harmful components, a challenge akin to the immune system’s struggle in pathogen detection. To address this issue, animals also rely on indirect sensing pathways to monitor food components [32].

Indirect sensing strategies are based on the associations between food components: a certain food component (the target) cannot be directly perceived, but it consistently coexists with another component (the proxy) that can be directly sensed [33]. For example, glutamate and ribonucleotides stimulate the appetite and promote the consumption of protein-rich foods by activating the umami receptors TAS1R1/TAS1R3 [34]. With experience, animals can associate the proxy with the target food component, enabling them to identify food. Although this proxy detection expands the range of identifiable food components, its specificity and sensitivity are constrained by the correlation between the target and the proxy.

Another indirect sensing strategy involves detecting the physiological effects of food components. Different food components can produce overlapping physiological effects, which makes this strategy less specific but more comprehensive. Specifically, food toxins can damage the integrity of cells or tissues, triggering alarm molecules (e.g., HMGB1 and IL-1α) to warn other cells of potentially harmful factors. Additionally, certain plant-derived secondary metabolites (e.g., enzyme inhibitors) can inhibit digestion or absorption, leading to the accumulation of unabsorbed nutrients in the gut, thereby triggering ileal motility and slowing down food transit through the digestive tract [35]. These mechanisms, although slower to respond, help animals react to potentially toxic components.

The sensitivity and specificity of sensory mechanisms are key features in this process. Typically, sensors for beneficial stimuli (such as glucose) exhibit low sensitivity but high specificity, leading animals to seek out high concentrations of nutrients. In contrast, sensors for harmful stimuli tend to have higher sensitivity but lower specificity, a mechanism that helps minimize false negatives but increases the likelihood of false positives. While a bias toward false positives aids in avoiding the ingestion of harmful substances, it may also lead to adverse food reactions [36,37].

As previously mentioned, the quality of food is determined by the relative amounts of its components, which can have positive, neutral, or negative effects on the organism. To evaluate overall food quality, the information detected by sensory mechanisms must be integrated with the organism’s physiological state and previous food experiences. This evaluation process is jointly executed by the nervous and immune systems and is far more complex than commonly assumed. Here, we primarily focus on the basic organizational structures that underpin these computational processes.

**Figure 1:**
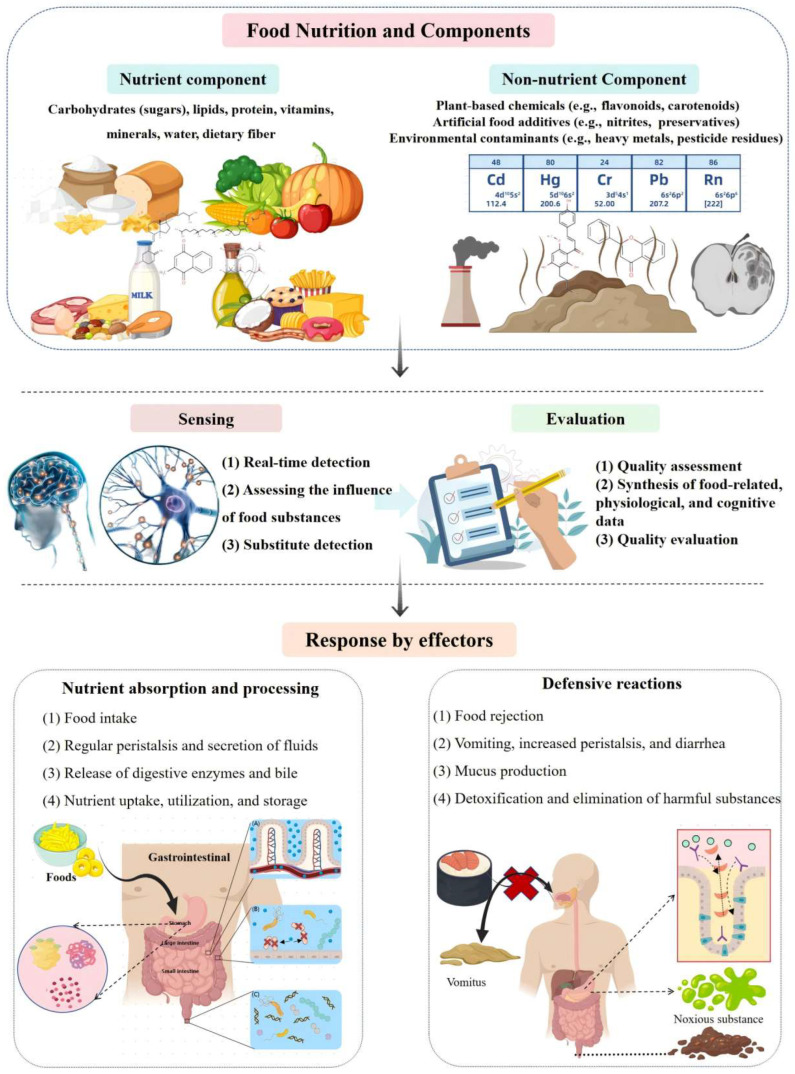
Food quality control process diagram: Food contains nutrients and other components that can be beneficial, neutral, or harmful. The animal assesses food quality by sensing these components directly, indirectly, or by detecting their physiological effects. This evaluation is influenced by the animal’s physiological state and past experiences. Based on the evaluation, the animal’s response may involve nutrient absorption processes (e.g., digestion and absorption) or defensive mechanisms (e.g., diarrhea and detoxification) to protect against harmful components. Partially cited from Created with MedPeer (https://medpeer.cn), URL (accessed on 20 November 2024).

The sensory information regarding food quality must be evaluated to determine the appropriate behavioral and physiological responses. For food evaluated as beneficial, animals will initiate responses that promote nutrient intake, digestion, and absorption. The response to foods with an overall negative value is the execution of various defenses (Figure 1). For food deemed harmful, animals will execute various defense mechanisms. Although the exact evaluation process remains unclear, both the immune and nervous systems play crucial roles in this process [38,39].

Harmful components in food may trigger intestinal inflammation and activate specific parts of the immune system, namely, the type II immune response regulated by T helper 2 (Th2) cells [33,40]. Since T cells require specific antigen peptides for activation, dietary proteins play a key role in initiating immune responses. Some dietary proteins may possess inherent toxicity, similar to toxins, but they often serve as substitutes for detecting harmful plant secondary metabolites (PSMs) [17]. In these cases, the negative effects of harmful chemicals in food are attributed to the protein antigens, thereby activating Th2 cells and antibody responses. Although T regulatory cells (Tregs) seem to play a dominant role in controlling the outcomes of immune responses, how the immune system assesses the “value” of food components remains not fully understood. In fact, in the absence of Tregs, animals and humans may experience severe immune reactions to dietary proteins, such as food allergies [41,42].

Furthermore, while the immune system assigns “value” to protein antigens, the nervous system assigns value to the sensory attributes of food and forms memories based on these sensory signals related to past experiences. For example, taste and smell are the primary means of food perception. The brain integrates external sensory information with internal physiological states, enabling adaptive physiological responses upon re-exposure. For instance, the taste of a food that previously made us ill not only triggers a rejection response but may also activate immune defenses in preparation for an imminent threat. Thus, the immune and nervous systems, through their respective mechanisms, integrate sensory inputs with past experiences to coordinate appropriate responses in an ever-changing environment [43].

## 3. Overview of Defense Mechanisms

Most foods contain potentially harmful low-quality components, and in the absence of appropriate defense mechanisms, the range of safely consumable foods would be severely restricted. Even small amounts of toxic food constituents can cause significant harm. To counter these risks, defense strategies can be classified into three main categories: avoidance, elimination, and adaptation. Avoidance relies on the early detection of potential threats, enabling the host to take proactive measures before exposure occurs, with behavioral avoidance playing a crucial role in detoxification defense. If harmful components are ingested, they can subsequently be neutralized or expelled. In contrast, adaptive strategies aim to mitigate the negative effects of low-quality components, often by preventing their absorption in the first place [44].

The anatomical structure of the digestive system facilitates a layered defense mechanism, operating across both time and space. These defenses can be categorized into three stages: pre-ingestive, post-ingestive, and post-absorptive. Pre-ingestive defenses primarily involve behavioral avoidance, while post-ingestive strategies function within the gastrointestinal tract itself. These strategies include digestive enzymes that neutralize harmful food components, neuronal reflexes that promote expulsion, and epithelial barriers that prevent these components from entering the body. Importantly, the immune system plays a critical role in coordinating post-ingestive defenses, ensuring an integrated and adaptive response. Post-absorptive defenses, on the other hand, involve the metabolism (detoxification) and excretion of potentially harmful food constituents. Furthermore, the gut microbiota provides an additional layer of defense through its ability to metabolize specific food components and modulate their effects [45].

**Figure 2:**
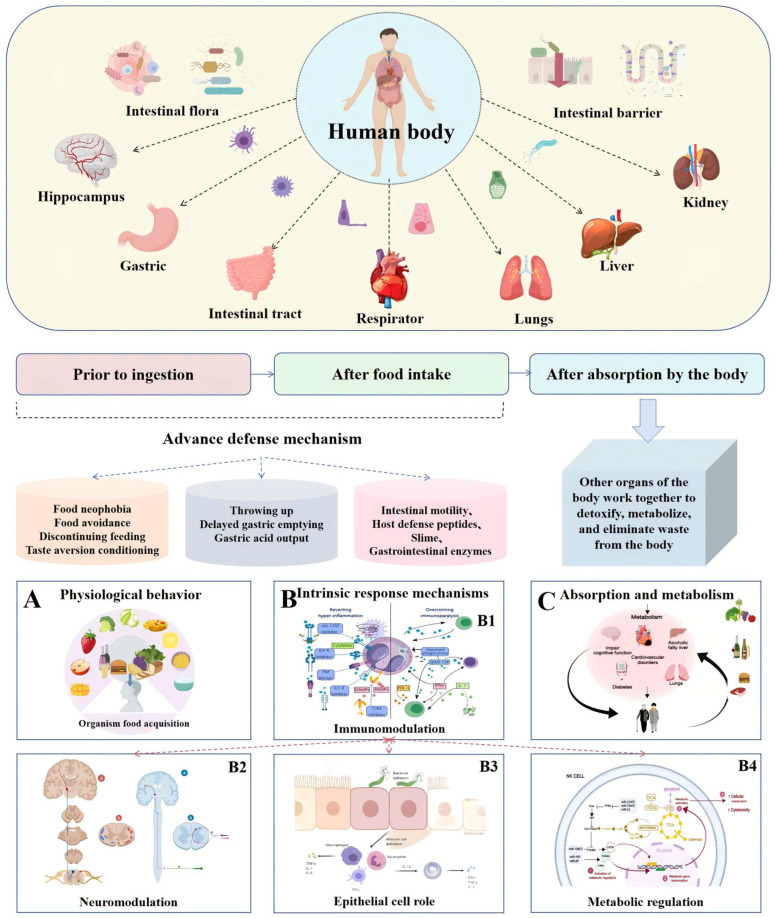
Role of all organs and related systems of the body’s defense mechanisms against viruses. The antitoxin component of the defense system contains several components that work in concert to promote the efficient absorption of nutrients and to protect the organism from, or mitigate the effects of, harmful food components by preventing, eliminating, or adapting to the presence of harmful substances. Partially cited from Created with MedPeer (https://medpeer.cn), URL (accessed on 20 November 2024).

These defense mechanisms often work synergistically, ensuring effective protection by complementing one another. Alternatively, they can compensate for one another when a particular strategy is insufficient or fails to activate. Nevertheless, it is essential to recognize that the operation of defense systems comes at the expense of host adaptability. Notably, defense against low-quality foods often conflicts with the process of nutrient assimilation [46]. For instance, while vomiting or diarrhea can expel ingested toxins, these processes also result in the loss of essential nutrients, thus preventing their absorption. More broadly, the costs of defense may arise from energy trade-offs, collateral damage associated with immune activation (immune pathology), or the suppression of other physiological functions that are incompatible with defense processes. Ideally, detoxification defenses should operate in an optimized combination and intensity, allowing for sufficient nutrient absorption while simultaneously protecting the host from harmful food components and minimizing associated costs. However, in some cases, defenses may be either inadequate, excessive, or misdirected, leading to compromised health outcomes (Figure 2).

### 3.1. Defensive Mechanisms in Feeding Behavior

To minimize the risk of ingesting potentially harmful substances, animals evaluate food at multiple pre-ingestive checkpoints. Initially, this evaluation is based on a combination of visual and olfactory cues, serving two primary purposes: first, to assess whether the food is familiar, and second, to detect any quality-related indicators, whether positive or negative [47]. When a food item is unfamiliar, the animal enters a heightened state of vigilance or suspicion, a phenomenon known as “neophobia”. This strategy is thought to reduce the likelihood of consuming harmful substances by temporarily decreasing appetite and enhancing alertness during the evaluation process. For instance, colors like blue-green or odors associated with bacterial growth, such as specific amines, signal food spoilage, triggering rejection. This olfactory evaluation occurs through complex interactions between olfactory sensory neurons and the olfactory receptor cells they innervate. If these sensory assessments are deemed acceptable, the animal proceeds with ingestion [48,49].

Once the food enters the mouth, it undergoes further evaluation, primarily through taste and texture. Certain textures, such as creaminess or smoothness, are often indicative of higher fat content, suggesting a higher caloric density, and thus promoting ingestion. In contrast, substances that induce discomfort, such as oral irritation or pain, trigger rejection [50]. While the exact mechanisms behind these sensations remain incompletely understood, it is believed that they are assessed through the fine mechanosensory and chemical detection systems of the trigeminal and facial nerves. Taste, on the other hand, relies on specialized sensory epithelial cells within taste buds, which are innervated by specific branches of the facial, glossopharyngeal, and vagus nerves [51]. These taste receptor cells express molecular profiles specific to the detection of particular taste compounds and, similar to olfactory receptors, are connected to sensory neurons that project to various brain regions, generating specific taste perceptions. The five primary tastes, sweet, salty, sour, bitter, and umami, reflect the activation of these specialized taste cells. For instance, saltiness is detected through Na^+^ channels, while sweetness and umami are mediated by distinct taste receptors that bind to molecules signaling nutrient availability, such as T1R2/T1R3 receptors for sugars and T1R1/T1R3 receptors for glutamate [52,53].

Within normal physiological ranges, sweet, salty, and umami flavors are considered appetitive, while sourness is tolerable only within a narrow range, and excessive sourness is rapidly avoided. Of all the tastes, bitterness is typically the most aversive. Bitter compounds are detected by T2R genes, a family of approximately 30 distinct bitter G-protein-coupled receptors expressed in bitter receptor cells [54,55]. These receptors are thought to evolve to detect molecular features associated with toxins. For example, the T2R14 receptor detects microtoxins, such as those found in the potent plant-derived neurotoxin from Anamirta cocculus [56].

Although pre-ingestive defenses serve to minimize the likelihood of ingesting toxic foods, this system is not infallible, and additional layers of defense are activated post-ingestion. These post-ingestive mechanisms are coordinated through several sensory pathways connecting the gastrointestinal tract to the brain, including the dorsal root ganglion spinal nerves, the vagus nerve, enteric neurons, visceral nerves, and blood-borne signals that affect brain regions like the hindbrain. These post-ingestive pathways frequently reinforce (or negate) the information conveyed by earlier sensory cues [57]. For example, ingested toxins may directly or indirectly stimulate serotonin release from enterochromaffin cells in the gut. This serotonin can bind to vagal nerve cells expressing the 5HT3 receptor, resulting in the acute cessation of food intake, even if the food was initially perceived as palatable [58]. Additionally, resident immune cells in the gut, such as mast cells, may activate similar pathways to halt ingestion when allergens or harmful substances are detected [59].

Although these responses provide a sensitive monitoring system to assess food quality and drive immediate decisions to accept or reject food, past experiences with food significantly influence future choices. Many pathways that strongly inhibit food intake also promote the formation of intense negative hedonic memories, a phenomenon known as conditioned taste aversion (CTA), when taste serves as the stimulus. As discussed, this strategy exemplifies proxy detection, in which sensory characteristics (e.g., taste or odor) of specific foods are associated with potential harm. The accuracy of this system is optimized under several conditions that enhance the reliability of the associations. For instance, novel foods are more likely to trigger CTA than familiar ones, and the temporal proximity of the negative stimulus to the taste reinforces the strength of the association [60]. Sensory cues related to food, such as smell and taste, are far more likely to form negative food memories than other cues, such as auditory or visual signals. Consequently, this system ensures that foods that have previously caused illness are less likely to be ingested in the future [61].

An important aspect of pre-ingestive sensory recognition is its role in preparing the body for the type of food to be consumed, a phenomenon known as the cephalic phase of feeding. For example, as originally described by Ivan Pavlov, the mere sight or smell of a favored food can trigger salivation and gastric acid secretion, which aids in digestion. Interestingly, recent studies suggest that this cephalic phase may also extend to immune defenses [62]. Previous sensory experiences associated with a particular immune challenge can lead to the establishment of immune memory in the nervous system, meaning that sensory recognition alone can alter immune responses. While the exact mechanisms, scope, and breadth of this phenomenon remain unclear, it is reasonable to hypothesize that this mechanism serves to prime the organism for anticipated harmful challenges, enabling more rapid and effective defense responses when needed.

### 3.2. Defensive Mechanisms After Ingestion

Once food is ingested, several interacting components come into play, optimizing nutrient absorption while simultaneously protecting the host from harmful food constituents. The digestive process, beginning in the oral cavity and continuing through the stomach and small intestine, not only facilitates nutrient absorption but also plays a crucial role in defense mechanisms. Gastric acid, secreted by stomach lining cells, serves a dual purpose: it breaks down food, denatures toxins and irritants, and neutralizes certain pathogens [63]. In addition, the vagus nerve’s neuronal reflex enhances this protective response by delaying gastric emptying and increasing acid secretion upon detecting toxic threats [64]. Moreover, digestive enzymes, produced and activated collaboratively by the salivary glands, stomach, pancreas, and small intestinal epithelial cells (such as amylase, trypsin, and pepsin), continue to break down food into absorbable components.

Furthermore, a common post-ingestive defense mechanism is the expulsion of low-quality, toxin-laden food through vomiting or diarrhea, a coordinated reflex mediated by the autonomic and enteric nervous systems. Diarrhea, in particular, is often the natural result of osmotic substances entering the colon, which may include undigested food. Consequently, in cases of food maldigestion, diarrhea also serves as a compensatory defense mechanism, ensuring the rapid removal of harmful substances.

In parallel, the intestinal epithelial cells (IECs) and their secretory components form a critical intestinal barrier. This barrier selectively absorbs nutrients and maintains fluid balance while simultaneously protecting the gut from harmful substances. The IECs are connected by protein complexes that form tight junctions, limiting the paracellular transfer of toxins and ensuring the integrity of the barrier, even as IECs turnover [65]. Notably, specialized IECs, such as goblet cells, secrete mucin, thereby creating an additional physical barrier between the lumen and the host. In cases of chronic toxin exposure, these cells undergo remodeling, supporting the proliferation of goblet cells and thereby enhancing the mucosal barrier. Beyond their secretory functions, these and other specialized IECs, such as M cells, enable the selective transport of lumenal substances, such as food antigens, across the immune system recognition barrier, facilitating the development of immune responses to harmful food components [66].

Moreover, immune cells play a prominent role in post-ingestive defenses. Upon activation, mast cells release various inflammatory mediators, including histamine and leukotrienes, which act on IECs, enteric neurons, and smooth muscle cells, thereby inducing diarrhea. Additionally, cytokines secreted by intestinal lymphocytes guide the remodeling of IECs in response to harmful stimuli [67]. As previously discussed, immune cells and mediators involved in the type II immune response are integral to toxin defense.

Importantly, during this defensive phase, a key trade-off exists between nutrient absorption and barrier protection. In most instances, the former comes at the expense of the latter, as mechanisms like accelerated expulsion, increased mucus production, and immune cell activation, especially when excessive, can impair nutrient absorption and lead to collateral tissue damage. Therefore, while the body defends against harmful food components, these responses can come at a cost, ultimately influencing overall nutritional status and health [68].

### 3.3. Defensive Strategies Following Food Ingestion

The absorption of food components from the gastrointestinal tract into the systemic circulation represents a pivotal transition in the interaction between an organism and the food it consumes. Once food components cross this threshold, host tissues are directly exposed to these substances, thereby making them susceptible to their potential effects. On the one hand, this process enables the host tissues to utilize or store nutrients from the diet according to physiological needs. On the other hand, the influx of food components into the circulatory system presents a serious challenge to internal homeostasis. This is because such exposure may not only make the host tissues vulnerable to toxic foreign organisms or pathogens but also risk overloading the body’s tolerance limits. Even essential nutrients, such as glucose, can exert adverse effects when present in excess. Therefore, to mitigate these risks during the post-absorptive phase, organisms have evolved a variety of metabolic defense mechanisms aimed at chemically modifying and/or eliminating harmful food components [64,69].

One of the key strategies employed by the organism involves biotransformation and detoxification processes. These terms refer to the enzymatic chemical modification of xenobiotics, foreign compounds introduced via food. These processes, which primarily occur in the gut and liver, have a protective role by reducing the toxicity of these substances and increasing their water solubility, thereby facilitating their excretion via urine [66]. Notably, many xenobiotics first undergo phase I reactions, which are mediated by enzymes like cytochrome P450 (CYP450) monooxygenases. These enzymes introduce functional groups through oxidation, reduction, or hydrolysis. Subsequently, in phase II reactions, these compounds are conjugated with hydrophilic groups, such as glutathione, further enhancing their solubility. Importantly, these enzymes are typically upregulated when the body is exposed to specific xenobiotics. This regulation occurs through xenobiotic sensors, such as the pregnane X receptor (PXR), constitutive androstane receptor (CAR), and the aryl hydrocarbon receptor (AHR), which enables the detoxification system to be induced as required [70]. However, it is crucial to recognize that while these reactions generally reduce the toxicity of foreign compounds, there are cases where chemical modifications can paradoxically increase their toxicity. For instance, plant-derived pyrrolizidine alkaloids can be metabolized by CYP450 enzymes into reactive intermediates that bind to essential macromolecules, such as proteins and nucleic acids, leading to cellular damage [71]. This harmful side effect underscores the potential costs associated with detoxification mechanisms.

Furthermore, excretion plays a fundamental role in eliminating both exogenous and endogenous waste products, including those derived from food intake. Xenobiotics and waste are primarily eliminated through the kidneys into urine or through the liver into bile and subsequently feces. When xenobiotic compounds possess hydrophilic properties, their elimination via the kidneys becomes more efficient, a process that can be enhanced by detoxification mechanisms. In the kidneys, exogenous substances can be passively filtered through the glomerulus, while some compounds are actively secreted into the urine via transporters, including members of the organic anion transporter family. In contrast, in the liver, exogenous compounds are actively transported into bile via ATP-binding cassette (ABC) transporters. Unlike renal excretion, biliary excretion is primarily limited to large, polar compounds (>300 g/mol) with lipophilic groups [72,73]. These elimination pathways are vital not only for removing harmful exogenous substances but also for clearing endogenous waste products, such as urea and creatinine, which are by-products of normal metabolic processes, as well as excess nutrients like glucose. Collectively, these pathways ensure that the exposure of host tissues to potentially harmful substances is minimized, thereby maintaining internal homeostasis.

Importantly, the efficiency of these detoxification and excretion mechanisms can vary significantly between species. For instance, dogs are more sensitive to theobromine, a toxic alkaloid found in chocolate and tea, than humans [74]. This difference is primarily attributed to variations in the metabolism and elimination rates of theobromine between species. In humans, the half-life of theobromine is approximately 2–3 h, whereas in dogs, it can be as long as 18 h. The prolonged half-life in dogs is thought to be due to extensive enterohepatic circulation, where theobromine is excreted into the intestines via bile, only to be reabsorbed into circulation, thereby delaying its eventual elimination. Another example involves digoxin, a cardiac glycoside found in foxglove leaves, which is detoxified by P450 IIIA enzymes. Therefore, species with higher P450 IIIA activity tend to exhibit lower sensitivity to digoxin [75,76]. Thus, the variation in metabolic defense capabilities across species reflects the evolutionary adaptations to specific dietary toxins encountered in their ecological niches. Notably, many of these toxins, such as theobromine and digoxin, are plant-derived, further emphasizing the role of plant-based foods as a major driving force behind the evolution of detoxification mechanisms.

While much remains to be understood about the immune system’s role in post-absorptive defense against food toxins, it is reasonable to hypothesize that immune-mediated mechanisms contribute additional layers of protection. For instance, immune responses associated with allergic reactions may alter blood flow, as evidenced by histamine-induced vasodilation in smooth muscle and endothelial cells, which could influence the distribution of toxins and protect vital tissues [77]. Moreover, food-specific antibodies may neutralize toxins or facilitate their sequestration and elimination. Immune cells also secrete enzymes, such as glycosylating enzymes and proteases, which may aid in detoxifying food toxins that are susceptible to these enzymatic modifications. Additionally, immune cells may regulate the activity of classical detoxification and excretion pathways, mediated by the kidneys, liver, and intestines, through cytokine signaling. Given the complexity of these processes, future research should explore these immune-mediated mechanisms in greater detail to better understand their contribution to post-absorptive defense [78].

### 3.4. Microbiota Modulation Mechanisms After Ingestion

In nearly all animals, the gastrointestinal tract harbors diverse microbial communities (microbiota) that influence many aspects of physiology. Their location enables direct interaction with both ingested food components and host tissues. While comprehensive discussions on the structure, products, and functions of microbiota communities have been extensively reported elsewhere [79], which falls beyond the scope of this review, we offer two general observations regarding their involvement in post-ingestion responses.

First, microbiota metabolism of food components can alter the overall quality of the food. In their beneficial role, microbiota can increase the total nutritional yield of food by digesting host enzymes’ hard-to-digest, nutrient-rich substances. For instance, animals consuming plant-based diets typically harbor microbiota capable of digesting plant-derived non-starch polysaccharides (e.g., cellulose and inulin), thereby releasing monosaccharide units (such as glucose) that can be utilized by the host [80]. Moreover, microbiota can isolate or metabolize foodborne toxins, thus protecting the host from their harmful effects [81]. Conversely, microbiota metabolism can also deplete or otherwise eliminate nutrients or even convert non-toxic substances into toxins or toxin precursors, thus negatively impacting overall food quality. For example, certain gut microbes can convert the dietary nutrients choline and L-carnitine into trimethylamine (TMA), which is subsequently absorbed by the liver and converted into trimethylamine-N-oxide (TMAO), a compound implicated in endothelial damage and atherosclerosis.

Second, microbiota metabolism of food components can generate signaling molecules that influence host defense mechanisms. This phenomenon has been particularly well-studied within the immune system. For example, short-chain fatty acids (SCFAs) produced by colon bacteria through fermentation of dietary fibers can directly regulate immune cell function by activating G protein-coupled receptors such as GPR41, GPR43, GPR109A, or OR51E2, or by inhibiting histone deacetylases [82]. Through these mechanisms, SCFAs derived from the microbiota typically dampen immune responses, for instance, by promoting the differentiation of regulatory T cells (Tregs). Another example involves the microbial conversion of dietary tryptophan into serotonin, indole, and related compounds, which act as ligands for the aryl hydrocarbon receptor (AHR), a nuclear receptor expressed in many cell types [83]. Tryptophan metabolites that activate AHR influence a range of defense pathways, particularly those involving the immune system, epithelial barriers, and detoxification pathways. These examples constitute only a small portion of the microbiota metabolome, which has the potential to impact host responses to food. However, much remains unknown about the full extent of these effects [84].

### 3.5. Ecological Perspective on Defense Mechanisms

The health benefits and costs of a specific set of defensive mechanisms depend on the dietary environment in which they operate. Consequently, the benefit-to-cost ratio of one group of foods may be very high, while that of another may be quite low. Evolution has shaped defenses, as well as the trade-offs between defense and nutritional processing, optimizing these mechanisms for the specific food sets available within an animal’s native ecological niche. For example, koalas possess detoxification pathways that are highly effective in eliminating toxic secondary metabolites, such as monoterpenes, which are derived from their primary food source, the eucalyptus leaves [85]. However, it is postulated that these defenses may not perform as effectively against many other plant-based foods that koalas did not encounter during their evolutionary history. They may be unable to defend against certain plant toxins or might respond inappropriately to some non-toxic substances. Thus, changes in an animal’s nutritional environment may result in a mismatch between evolved defenses (and, more broadly, nutritional strategies) and the available food, potentially leading to a higher incidence of adverse food reactions due to suboptimal defense responses. These reactions can manifest as acute responses (e.g., vomiting or diarrhea shortly after ingesting problematic foods) or chronic effects (e.g., chronic inflammation or pathology of unknown etiology after prolonged consumption of problematic foods) [86].

In order to understand why certain types of food-related adverse reactions are becoming increasingly prevalent in modern society, it is essential to first consider the significant changes in human dietary patterns over recent history and their effects on our physiology. Beginning approximately 2.5 million years ago, our ancestors lived as hunter–gatherers, subsisting on foods derived from wild plants and animals. Their diet was highly diverse, varying by region and season, and typically included fruits, nuts, seeds, plant storage organs (e.g., tubers), honey, and meat [87]. The advent of cooking (at least 780,000 years ago) increased the digestibility of various foods, particularly meats, thereby enhancing their nutritional yield. This versatile nutritional strategy remained dominant until the first agricultural revolution, which began around 12,000 years ago. This revolution replaced the diversity of the hunter–gatherer diet with a relatively small number of domesticated plants, most notably grains such as wheat, barley, and rice, as well as domesticated animals [88]. Due to historical and geographical factors, the specific combination of domesticated crops and animals varied around the world, but the reliance on a relatively small number of staple foods was a common theme.

The transition to agriculture resulted in a more abundant and stable food supply, enabling population growth and the development of human civilizations. However, the increase in food production came at the expense of nutritional quality. Compared to the traditional hunter–gatherer diet, to which our physiological systems are well adapted, the modern grain-based diet typically contains lower concentrations of protein, fiber, micronutrients, and other beneficial components [89]. This loss in nutritional quality can even be observed within the same crop family. For instance, modern wheat varieties, such as common wheat, have lower protein and mineral content compared to ancient wheat species like einkorn and emmer, reflecting the evolutionary changes that occurred during wheat domestication [90]. This difference is further amplified by modern food processing practices, which often remove the nutrient-rich portions of plants (e.g., the germ and bran during the refining of grains). The addition of sugars, fats, and salts increases the calorie density and/or palatability of modern foods, encouraging consumption even when certain nutrients are relatively deficient. Furthermore, artificial chemicals are introduced at various stages of food production to enhance yield, preservation, or other desirable characteristics (e.g., flavor, texture), many of which may have harmful effects on human health, some of which remain poorly understood [91]. Over time, the combination of nutrient deficiencies and harmful food additives may impair physiological processes, including defense responses, thus increasing the risk of adverse food reactions.

## 4. Conclusions

Despite the growing body of research on the toxicity of ingested foods, our understanding of the mechanisms underlying food adverse reactions and their treatments remains incomplete. The food quality control framework proposed in this article helps to elucidate these reactions in relation to normal physiological processes. Multiple systems enable organisms to coexist with symbiotic microbes, regulate them, and simultaneously avoid, expel, or neutralize harmful invading pathogens. Similarly, food quality control systems allow organisms to absorb the necessary nutrients while defending against low-quality or harmful components in food. These systems involve mechanisms for sensing food components, evaluating food quality, and directing appropriate defensive responses to avoid, expel, or adapt to harmful food constituents.

Although many microbes can be lethal in the absence of antimicrobial defenses, there has been a noticeable rise in diseases related to microbiome dysregulation, such as inflammatory bowel disease. In a similar vein, antitoxin defenses are not without their costs, and they may fail to function optimally due to insufficiencies, exaggerations, or misdirected actions, ultimately leading to various adverse food reactions. It is highly likely that changes in human dietary and lifestyle patterns over recent history have increased the occurrence of defense failures, thus contributing to the rising incidence of food-related adverse reactions. While it is plausible to hypothesize which defensive failures underlie modern food intolerances, experimental insights into the root causes of many of these reactions remain elusive, with a lack of suitable biomarkers to evaluate antitoxin defenses. In the future, a more comprehensive understanding of food quality control mechanisms will be a crucial step in developing more effective treatments for food-triggered diseases.

## Figures and Tables

**Table 1:** The beneficial effects of plant nutrients and the toxicity of their secondary metabolites.

Foods	Samples	Mechanisms of Action	References
	The disadvantages of non-nutrient secondary metabolites (blocking the absorption of nutrients)		[22,23,24,25,26,27,28]
Wheat, maize, rice, tomatoes, oats, barley	α-Amylase inhibitors, trypsin inhibitors, pancreatic lipase inhibitors, α-glucosidase inhibitors, cellulase inhibitors	Affects nutrient absorption during digestion mainly by interfering with the catalytic activity of enzymes.	
Beans, seeds, nuts, whole grains, leafy greens, vegetables	Tannins, phytic acid, cyanogenic glycosides, oxalates	Can bind with nutrients to form insoluble complexes, leading to the precipitation of nutrients.	
Legumes (e.g., pisum sativum, glycine max, etc.), pseudo-legumes (e.g., chickpeas, millet, etc.)	Lectins (e.g., ConA, PHA), toxins, non-starch polysaccharides	Disrupt epithelial function, especially in gut health.	
	The advantages of nutritional components (enhance the body’s functions)		
Wheat, maize, rice, tomatoes, oats, barley, beans, seeds, nuts, whole grains, leafy greens, vegetables, legumes, pseudo-legumes	Dietary fiber: Improves gut health and prevents constipation. Protein: Provides essential amino acids, aiding muscle repair and immune function. Essential fatty acids: Support brain and heart health; Starch: Provides sustained energy. Vitamins (A, C, E, K, B group): Enhance immunity, act as antioxidants, and support bone health and energy metabolism. Minerals (calcium, iron, magnesium, potassium, zinc): Strengthen bones, promote blood cell production, and regulate nerve and heart function. Isoflavones: Balance hormones; Phytates: Antioxidant properties may reduce cancer risk. Phenolic compounds: Anti-inflammatory and antioxidant effects. Insoluble fiber: Promotes bowel movement.		
	The disadvantages of non-nutrient secondary metabolites (impaired cell function or dysregulation of physiological processes)		
Legumes (e.g., soybeans, peas, lentils, fava beans)	Saponins	Disruption of cell membranes, leading to gut cell damage, increased intestinal permeability, and potential digestive issues and immune responses.	
Alliums (e.g., garlic, onion, leeks)	Sulfur compounds (e.g., allicin)	Disruption of cell membranes and interference with enzyme activity, potentially irritating the gastrointestinal tract and affecting digestive system function.	
Cruciferous Vegetables (e.g., broccoli, cabbage, cauliflower, mustard greens)	Glucosinolates (e.g., sinigrin, glucoraphanin)	Damage to biomolecules such as proteins and DNA, possibly causing gastrointestinal discomfort and, with long-term high intake, affecting thyroid function.	
Spices and herbs	Monoterpenes (e.g., limonene, menthol)	Unknown mechanism; may irritate the digestive tract, potentially disrupting gut health and metabolism.	
Bitter almonds (e.g., bitter almonds, certain nuts like almonds, walnuts)	Cyanogenic glucosides (e.g., amygdalin, prunasin)	Release of cyanide, inhibiting mitochondrial respiration, leading to energy metabolism disruption, potentially causing poisoning symptoms such as nausea, headaches, and difficulty breathing.	
Sorghum, cassava, lima beans	Cyanogenic glucosides (e.g., linamarin)	Release of cyanide, inhibiting mitochondrial respiration, leading to mitochondrial dysfunction, poisoning, and respiratory and neurological symptoms.	
Cacao, coffee, tea	Alkaloids (e.g., caffeine, theobromine, theophylline)	Interference with signaling pathways in the central nervous system, leading to stimulation, insomnia, increased heart rate, and anxiety.	
Legumes (e.g., soybeans, peas, fava beans, chickpeas)	Nonprotein amino acids (e.g., canavanine)	Interference with protein synthesis, leading to immune system dysfunction and potentially autoimmune responses or cellular damage.	
Maize, wheat, rye	Benzoxazinoids (e.g., dimboa, hdmbboa)	Unknown mechanism; may negatively impact gut microbiota, leading to digestive discomfort or increased intestinal permeability.	
Certain fruits (e.g., plums, cherries, grapes)	Cyanogenic glucosides (e.g., amygdalin, cherry pits)	Release of cyanide, leading to mitochondrial respiration inhibition, respiratory distress, and neurological symptoms.	
Pumpkin seeds, tomato seeds	Alkaloids (e.g., solanine)	Disruption of cell membrane integrity, leading to gastrointestinal irritation and potentially causing nausea, vomiting, or diarrhea.	
Nightshade plants (e.g., potatoes, eggplants, peppers)	Alkaloids (e.g., solanine, tropane alkaloids)	Interference with the nervous system, causing symptoms such as headaches, nausea, vomiting, and, in severe cases, neurological effects.	
	The advantages of nutrients (improvement of body immunity and function)		
Legumes, alliums, cruciferous vegetables, spices and herbs, bitter almonds, sorghum, cassava, lima beans, cacao, coffee, tea, legumes, maize, wheat, rye, certain fruits, pumpkin seeds, tomato seeds, nightshade plants	Plant protein: Legumes and maize support muscle growth and repair. Sulfur compounds: Allium plants have anti-inflammatory and antibacterial effects. Carotenoids: Lycopene in tomatoes protects eyes with antioxidant properties. Flavonoids: Coffee and tea flavonoids promote antioxidant and cardiovascular health. Alkaloids: Cocoa alkaloids improve mood and cognition. Tannins: Coffee and tea tannins aid antioxidant activity and digestion. Dietary Fiber: Maize, almonds, and cassava support gut health. Polyphenols: Tea and coffee polyphenols reduce aging and oxidative stress. Magnesium: Cassava and legumes support nerve and muscle function. Folic Acid: Legumes and leafy greens support cell repair. Amino Acids: Legumes and nuts aid tissue repair and immune function.		

Abbreviations: ConA, concanavalin A; PHA, phytohemagglutinin; DIMBOA, 2,4-dihydroxy-7-methoxy-(2H)-1,4-benzoxazin-3(4H)-one.

## Data Availability

No data were used for the research described in the article.
